# Age structure and body size of two Tibetan toad (*Bufo tibetanus*) populations from different elevations in China

**DOI:** 10.1002/ece3.11559

**Published:** 2024-06-11

**Authors:** Mengshuang Xu, Gege Wang, Putong Liu, Zhuolin He, Kaiqin He, Zhiqiang Cheng, Ziqi Wang, Wei Chen, Zhibing Li, Lixia Zhang

**Affiliations:** ^1^ Department of Ecology, College of Life Sciences Henan Normal University Xinxiang Henan China; ^2^ School of Resources and Environmental Engineering Anhui University Hefei Anhui China; ^3^ The Observation and Research Field Station of Taihang Mountain Forest Ecosystems of Henan Province, Puyang Field Scientific Observation and Research Station for Yellow River Wetland Ecosystem and Research Center for ecological management and protection of the Yellow River Basin Henan China

**Keywords:** age, body size, *Bufo tibetanus*, elevation effect, skeletochronology

## Abstract

Understanding how age and body size vary across elevations can provide insights into the evolution of life‐history traits in animals. In the present study, we compared the demographic (using skeletochronology) and morphological traits of the Tibetan toad (*Bufo tibetanus*) between two populations from different elevational habitats (2650 vs. 3930 m). We found that (1) the mean age and body size of females were significantly greater than those of males in both populations; (2) both sexes of toads from the higher elevation tended to be significantly older in age and larger in body size; (3) there was a significant positive relationship between age and body size within each sex of the toad at both elevations; and (4) growth rates varied between the two populations, with the higher rate observed in the lower‐elevation population. Our results suggested that factors other than age, such as elevation‐associated temperature, influence the observed differences in body size between the two populations. Future research at a broader range of elevations should focus on these factors and evaluate their influence on animal growth patterns.

## Graphical abstract

Life‐history traits, such as age and body size, can evolve in response to climate variations along elevational gradients. We found the Tibetan toad (*Bufo tibetanus*) exhibits a slow life history at high elevations, with females being older and larger than males, and individuals from higher elevations being older and larger than those from lower elevations. This suggests that factors other than age, such as elevation‐associated temperature, can influence latitudinal patterns in body size.

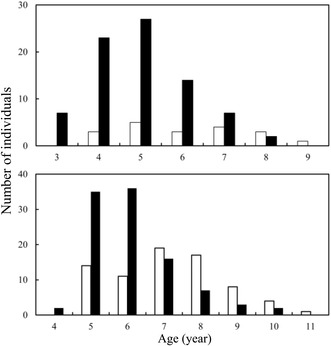

## 1 INTRODUCTION

Body size is a key feature of all organisms, and is closely related to fitness traits, including survival and reproduction (Roff, 2002). Body size variation, especially along geographical gradients, has been of great interest to ecologists, the most prominent of which is Bergmann's rule, which predicts that populations within a species or closely related species tend to have larger body sizes at higher elevations or latitudes (Bergmann, 1847; Mayr, 1956). The underlying mechanism is primarily related to thermoregulation; that is, larger individuals have lower body area‐to‐volume ratios, enabling them to retain heat more efficiently and maintain stable body temperatures in cold environments, such as high‐elevation or high‐latitude regions (Olalla‐Tárraga & Rodríguez, 2007; Walters & Hassall, 2006). This rule has been described in many endothermic vertebrates (Birds: Ashton, 2002; Olson et al., 2009; Mammals: Ballinger & Nachman, 2022; Clauss et al., 2013; DeVries et al., 2022; McQueen et al., 2022; Mori et al., 2019). However, whether ectothermic vertebrates such as amphibians, which rely primarily on heat from the external environment, follow this rule remains controversial (Deme et al., 2023; Khatiwada et al., 2019; Yu et al., 2019).

The responses of amphibian life history traits to geographic variations are complex, especially elevational variations (Liao et al., 2016). Beyond low temperatures at high elevations, organisms contend with a combination of environmental factors, including increased seasonality, intense solar radiation, reduced atmospheric pressure, and limited oxygen availability (Körner, 2007). Thus, amphibians at high elevations may have low annual growth rates because of low metabolism, short growth seasons due to cold temperatures, low predation pressure, and long lifespans because of the low diversity of predators and long hibernation periods (Morrison & Hero, 2003). Indeed, many studies on amphibians have shown that elevation has great effects on many characteristics of living populations, such as body size, growth rate, maturation age, and longevity (Altunışık et al., 2022; Baraquet et al., 2018; Kalayci et al., 2015; Yu et al., 2022; Zhang & Lu, 2012). When examining the differences in the geographic patterns of life‐history traits, elevational comparisons within a species can offer valuable insights and provide complementary information for cross‐species comparisons. This is because (1) the breeding time and developmental patterns of amphibians are sensitive to environmental factors that show more elevation‐related trends at a fine scale (Duellman & Trueb, 1994; Wells, 2019), and (2) the influence of genetic variation is greatly reduced (Berven, 1982; Blanckenhorn & Demont, 2004).

Knowledge of life‐history traits (e.g., age and body size) evolve in response to climatic variation along elevational gradients, which is an important aspect of evolutionary biology (Guo et al., 2019; Laiolo & Obeso, 2017; Miaud et al., 2000; Morrison & Hero, 2003). Two methods, i.e., mark‐and‐recapture and skeletochronology, have been developed to obtain data on the age and growth of anurans. Gathering data using the former method requires considerable time and effort, as a large number of specimens is required because of the low recapture rate (Daugherty & Sheldon, 1982; Lehtinen, 2009; Smirina, 1994). In contrast, skeletochronology is a reliable method for estimating the age of amphibians by counting the lines of arrested growth (LAGs) in phalangeal cross‐sections (Peng et al., 2022; Sinsch, 2015). This technique enables researchers to collect information on demographic parameters (e.g., age, growth rate, and longevity) of the population in a relatively short time without killing the animals. This method has been used successfully in a variety of species of anurans, including species from temperate, subtropical, and tropical regions (temperate: López et al., 2017; Ma, Tong, & Lu, 2009; Zhang et al., 2013, 2020; Subtropical: Brum et al., 2019; Liao & Lu, 2010a; Zhou et al., 2010; tropical: Khonsue et al., 2000; Stanescu, 2015).

The reproductive investments of males and females are different; therefore, even in the same environment, they may experience different sexual selective forces, which can lead to differences in body size between the two sexes, a phenomenon known as sexual size dimorphism (Andersson, 1994; Fairbairn, 1997; Schäuble, 2004). Sexual size dimorphism is widespread; approximately 90% of anuran species exhibit female‐biased size dimorphism, with females having larger body sizes than males (Shine, 1979). In addition to body size, age at sexual maturity, longevity, and post‐metamorphosis growth rates may also be affected by different selective pressures between the sexes (Liao et al., 2015). Exploring how these sex‐specific traits vary across different environmental populations may provide further insight into sexual selection and Bergmann's rule.

The Tibetan toad, *Bufo tibetanus*, is endemic to southwestern China (Duellman, 1993; Duellman & Trueb, 1994; Frost, 1985). This species is distributed across a wide elevation range from 2400 to 4300 m above sea level (m asl) and is often found in grassland patches and farmlands (Fei et al., 2012). The toad may be classified as a prolonged breeder because of its breeding activity from April to July (Fei et al., 2012). Various studies have been conducted on its biology and ecology, including its mitochondrial DNA, skin structures, and male call characteristics (Cai et al., 2019; Gao et al., 2016; Wang et al., 2013). However, few studies have focused on populational age structure (except Li et al., 2022). The present study aimed to (1) investigate the age structure, body size, and growth patterns of two *B. tibetanus* populations inhabiting two extreme elevations of their distribution range (2650 vs. 3930 m asl), where environmental conditions differ greatly; and (2) compare age, body size, and growth traits between sexes within each population and between populations.

## 2 MATERIALS AND METHODS

### 2.1 Study sites and specimens

Toad samples were collected from wetlands close to two towns in southeastern Tibet, China. The lower‐elevation population is situated near Bomi town (29°53′N, 95°41′E, 2650 m asl), which is influenced by the Indian Ocean to the southwest. The study area is characterized by a humid subtropical climate, with a mean annual temperature of 8.7°C and annual precipitation of 799 mm (Li & Geng, 2018). This region is predominantly covered by dense evergreen broad‐leaved forests featuring alpine oak (*Quercus aquifolioides*), cypress, and birch tree (*Betula pendula*). The higher‐elevation population is located in Ranwu town (29°30′N, 96°45′E, 3930 m), about 110 km from the lower‐elevation population. The study area is characterized by a cold temperate plateau climate with sufficient sunshine. The mean annual temperature and rainfall are 2°C and 500 mm, respectively (Zhang et al., 2023). Under these climate conditions, land vegetation exists mainly as alpine meadows with dwarf shrubs and high alpine cushions.

During the breeding seasons of 2019 and 2022, 102 adult toads (19 females and 83 males) were caught by hand at the lower‐elevation site. In addition, 177 adult toads (76 females and 101 males) were caught at the higher‐elevation site during the same period. The snout‐vent (SVL) of each toad was measured with a digital Vernier caliper to the nearest millimeter, sexed according to secondary sexual characteristics such as nuptial pads in males and enlarged ova in females, and toe‐clipped on the second phalange of the longest finger of the right hind limb. All the clipped phalanges were stored in 5% neutral‐buffered formalin for skeletochronology. The toads were released in situ after the wounds were thoroughly disinfected with 75% ethanol using sterile cotton swabs.

### 2.2 Skeletochronology

The ages of the toads were estimated using skeletochronology as described by Ma, Lu, and Merilä (2009). The fixed phalanges were washed with tap water, decalcified in 5% nitric acid for approximately 48 h, and washed overnight under running tap water. Decalcified digits were stained with Ehrlich's hematoxylin for 100 min and rinsed with distilled water. Subsequently, the stained bones were dehydrated through an ethanol series (60 min each in 70%, 80%, 95%, and 100%) and infiltrated with successive paraffin changes for 1 h in an oven at 50°C and embedded in small paraffin blocks. The mid‐diaphyseal part of each phalange was cut into 12 μm‐thick transverse sections with a rotary microtome, and the sections with the smallest medullar cavity were examined using a Nikon MM‐40 microscope (Nikon, Tokyo, Japan). The best samples were photographed at the selected magnifications. Finally, two researchers observed and counted the LAGs under a light microscope.

Because the temperatures in these two study areas were markedly seasonal, the number of LAGs was a reliable indicator of toad age, as supported by the findings of most authors (Kumbar & Lad, 2017; Phadmacanty & Kurniati, 2019; Pollo et al., 2019). All specimens were collected during the breeding season, after emergence from hibernation; therefore, the outer margin of the bone was counted as an additional LAG. False lines, which may form during an unfavorable period in summer, are generally fainter than LAGs and do not form a complete ring around the bone section. We counted double lines in cross‐sections as 1 year of age; thus, they cannot affect the LAGs counted (Iturra‐Cid et al., 2010; Jiang et al., 2022; Khaloei et al., 2023; Phadmacanty et al., 2019; Yuan et al., 2021; Zhang et al., 2020).

### 2.3 Estimation of the growth rate

Once the age structure was estimated, we explored the population growth rates of females and males in both populations using the von Bertalanffy equation (von Bertalanffy, 1957). The equations are as follows:

$$\mathrm{SV}L_{t}=\mathrm{SV}L_{\max}-\left(\mathrm{SV}L_{\max}-\mathrm{SV}L_{0}\right)e^{-\mathrm{kt}},$$

where SVL_*t*_ = body size at age *t*, SVL_max_ = asymptotic maximum size, SVL_0_ = body size at metamorphosis (calculated as the mean SVL of the larvae captured at Gosner stage 42–46 in the two populations, and set at 16.5 mm for the lower elevation and 17.3 mm for the higher elevation), and *k* = growth coefficient. The mean growth rate was calculated as *R* = *k* (SVL_max_–SVL_mean_), where SVL_mean_ is the mean SVL of individuals in a given population.

### 2.4 Statistical analysis

Interpopulation and sex differences in age structure were assessed using chi‐square tests. When comparing mean ages, we used four generalized linear models (GLMs) to assess the inter‐sex differences for each population and intra‐sex differences for each sex, as age was a count variable. To check for possible variations in the effect of elevation on different sexes, we also conducted a GLM with sex, site, and a two‐way interaction term between them as independent variables with the pooled data. The GLMs all adopted a Conway–Maxwell–Poisson distribution and a log‐link function owing to the under‐dispersion in these models. Sex and site were treated as binary variables in all the analyses.

We first checked the normality for body size of each population and sex using the Shapiro–Wilk test and the results were significant, indicating that they were not normally distributed. Due to non‐normally distributed data, Mann–Whitney *U*‐tests rather than Student's *t*‐tests were used to compare body size between sexes or populations. Furthermore, we ran rank‐based regression models to test for sex or population differences in body size, with sex or population as binary predictor variables and age as a continuous covariate to exclude the effect of age on body size. We ran another rank‐based regression model with sex, population, and their interaction term as explanatory variables to test whether sex differences varied between the two populations.

We used Spearman's correlation test rather than Pearson's correlation test when exploring the relationship between age and body size in males and females from both populations, as body size did not increase linearly with age in either population.

All analyses were conducted in R version 4.3.2 (R Core Team, 2023) using the following packages: *glmmTMB* (version 1.1.8; Brooks et al., 2017) for GLMs, *Rfit* (version 0.24.6; Kloke & Mckean, 2012) for rank‐based regression models, and *nls.multstart* (version 1.3.0; Padfield & Matheson, 2023) for fitting the von Bertalanffy equation. All tests were two‐tailed with a significance level of α = 0.05.

## 3 RESULTS

### 3.1 Skeletochronology

**FIGURE 1:**
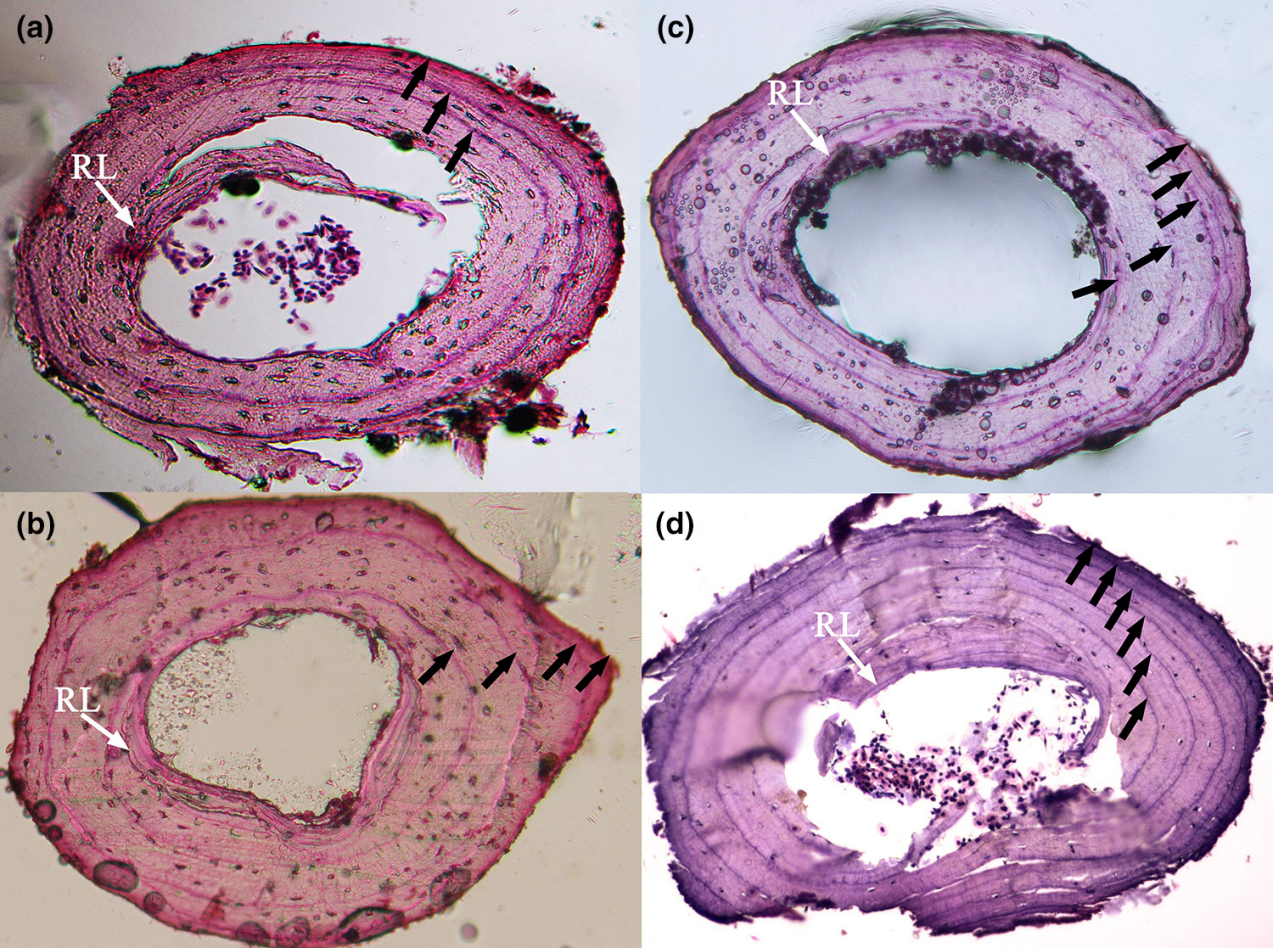
Four selected examples of hematoxylin‐stained cross‐sections of phalangeal bone of *Bufo tibetanus* from both populations in southwestern China. Low altitude, (a) a 5‐year‐old female, (b) a 7‐year‐old male; High altitude, (c) a 5‐year‐old female, (d) a 7‐year‐old male. Arrows indicate the LAGs, and RL represents Resorption Line.

LAGs were observed in the phalangeal sections of all toads except for five individuals (Figure 1); three males from the lower‐elevation population and two females from the higher‐elevation population lacked LAGs and were not included in the statistics. Double LAGs were observed in three males and four females in all samples. False LAGs were observed in two males and four females and were not considered true LAGs. Endosteal resorption was found in 14.91% of the 181 male samples and 11.83% of the 93 female samples. However, well‐defined Kastschenko lines (KLs) were observed in all samples, indicating that endosteal resorption caused no problems in age determination.

### 3.2 Age structure

**FIGURE 2:**
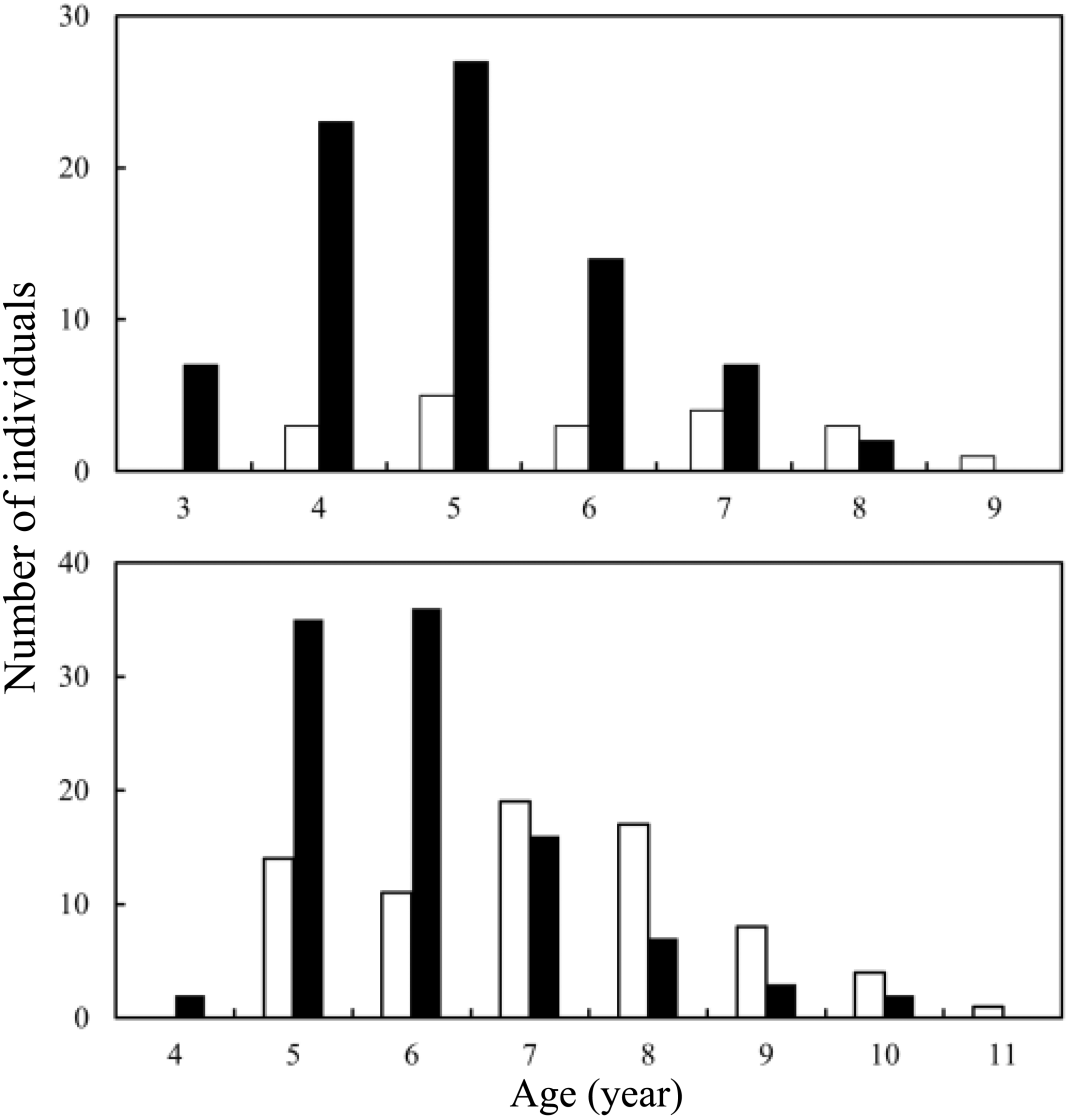
Adult age structure (male, close bars; female, open bars) of *Bufo tibetanus* for both high and low altitudes in southwestern China.

Age distributions in both populations showed significant differences between sexes (Chi‐square test: lower‐elevation, $\chi^{2}$ = 14.6, *df* = 6, *p* = .024; higher‐elevation, $\chi^{2}$= 29.2, *df* = 7, *p* < .001; Figure 2). In the lower‐elevation population, the predominant age class of both sexes was 5 years (female: 26.3%; male: 33.8%). The predominant age classes of females and males were 7 years (25.7%) and 6 years (28.0%), respectively, in the higher‐elevation population. The age distributions within each sex differed significantly between populations (Chi‐square test: female, $\chi^{2}$ = 14.4, *df* = 7, *p* = .045; male, $\chi^{2}$ = 44.9, *df* = 7, *p* < .001).

The GLM showed that females were significantly older than males in both populations (lower‐elevation: estimate ± SE = −0.207 ± 0.057, *z* = −3.66, *p* < .001; higher‐elevation: estimate ± SE = −0.160 ± 0.030, *z* = −5.28, *p* < .001; Table 1). The average longevity of both sexes differed significantly between populations (females: estimate ± SE = −0.156 ± 0.058, *z* = −2.67, *p* = .008; males: estimate ± SE = −0.202 ± 0.032, *z* = −6.35, *p* < .001; Table 1), with individuals at higher elevation being older than their counterparts at lower elevation. The interaction term between sex and site was not significant in the GLM model with pooled data (estimate ± SE = −0.047 ± 0.063, *z* = −0.75, *p* = .456), indicating that there was no variation between the two populations in terms of sex differences in mean age.

**TABLE 1:** Age and body size of two populations of the Tibetan toad *Bufo tibetanus* from southwestern China.

Sex	Low‐elevation population					High‐elevation population				
	Age (years)	Range	SVL (mm)	Range	*N*	Age (years)	Range	SVL (mm)	Range	*N*
Female	6.1 ± 1.5	4–9	54.6 ± 7.9	39.4–64.3	19	7.1 ± 1.5	5–11	71.6 ± 7.5	43.1–85.9	74
Male	5.0 ± 1.2	3–8	51.1 ± 4.2	40.3–63.0	80	6.1 ± 1.2	4–10	60.5 ± 5.0	39.9–70.5	101

*Note*: The values are given as the mean and the standard deviation, *n* = sample size.

### 3.3 Body size

The mean SVL differed significantly between sexes in the lower‐elevation population (females: 54.6 ± 7.9 mm; males: 51.1 ± 4.2 mm; Mann–Whitney *U*‐test: *Z* = 2.3, *n*_1_ = 19, *n*_2_ = 80, *p* = .02; Table 1). In the high‐elevation population, the mean SVL of females was also significantly larger than that of males (females: 71.6 ± 7.5 mm; males: 60.5 ± 5.0 mm; Mann–Whitney *U*‐test: *Z* = 9.3, *n*_1_ = 74, *n*_2_ = 101, *p* < .001; Table 1). The rank‐based regression models showed a significant difference in terms of SVL between sexes only in the higher elevation population when the effect of age was removed (lower‐elevation: estimate ± SE = −0.112 ± 0.805, *t* = −0.140, *p* = .889; higher‐elevation: *t* = −17.099, *p* < .001).

The mean SVL of females in both populations differed significantly (Mann–Whitney *U*‐test: *Z* = 6.0, *n*_1_ = 19, *n*_2_ = 74, *p* < .001), and the mean SVL of males differed significantly between populations (Mann–Whitney *U*‐test: *Z* = 9.8, *n*_1_ = 80, *n*_2_ = 101, *p* < .001). There was a significant difference in SVL between populations in both sexes when the effect of age was controlled, with toads from lower‐elevation population being smaller than those from higher‐elevation population (female: rank‐based regression, estimate ± SE = −13.557 ± 0.974, *t* = −13.912, *p* < .001; male: rank‐based regression, estimate ± SE = −6.826 ± 0.423, *t* = −16.155, *p* < .001). Moreover, the interaction term between sex and population was significant with pooled data, suggesting that sex difference in body size in the higher elevation was larger than that in the lower elevation (estimate ± SE = 7.483 ± 0.869, *t* = 8.612, *p* < .001).

### 3.4 The relationship between age and body size

**FIGURE 3:**
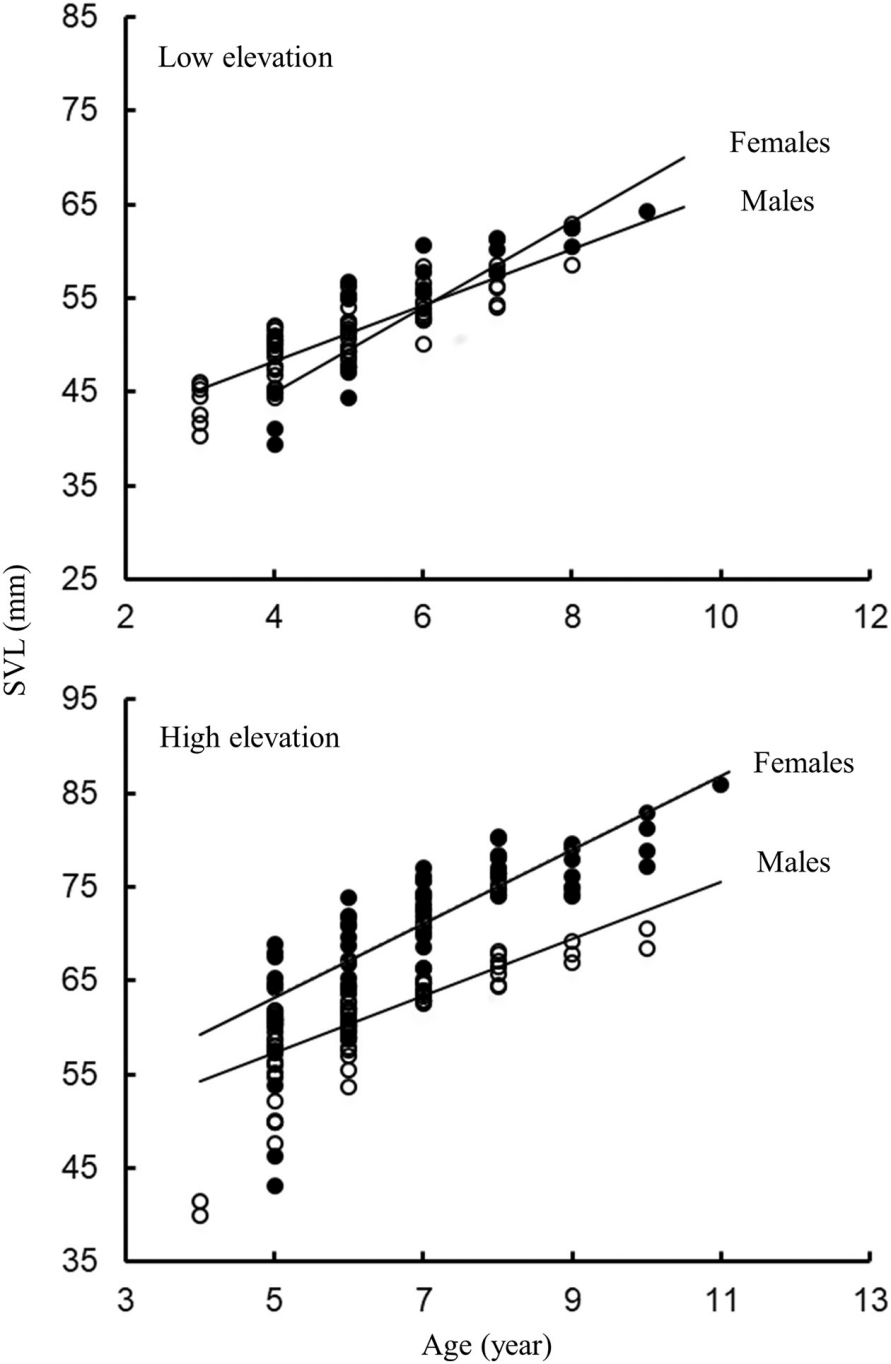
The relationship between age and body size of *Bufo tibetanus* (females, closed circle; males, open circle) for both sexes in southwestern China.

A positive correlation between age and body size was observed for both males and females in both populations (Spearman's correlation coefficient: lower‐elevation, females, *r*_*s*_ = .878, *n* = 19, *p* < .001; males: *r*_*s*_ = .834, *n* = 80, *p* < .001; higher‐elevation, females, *r*_*s*_ = .797, *n* = 74, *p* < .001; males, *r*_*s*_ = .733, *n* = 101, *p* < .001; Table 2, Figure 3).

**TABLE 2:** Body size (SVL) in relation to age in the Tibetan toad *Bufo tibetanus* from two populations in southwestern China.

Age class	Low elevation (2650 m)		High elevation (3930 m)	
	Females	Males	Females	Males
3		43.7 ± 2.2 (*n* = 7)		
4	41.8 ± 2.9 (*n* = 3)	49.1 ± 2.4 (*n* = 23)		40.7 ± 1.1 (*n* = 2)
5	50.2 ± 5.4 (*n* = 5)	51.1 ± 2.3 (*n* = 27)	60.4 ± 7.9 (*n* = 14)	58.0 ± 3.9 (*n* = 35)
6	58.2 ± 2.4 (*n* = 3)	54.1 ± 2.0 (*n* = 14)	69.2 ± 3.1 (*n* = 11)	60.3 ± 2.4 (*n* = 36)
7	59.2 ± 1.9 (*n* = 4)	57.0 ± 2.5 (*n* = 7)	72.5 ± 2.8 (*n* = 19)	63.9 ± 1.1 (*n* = 16)
8	61.8 ± 1.2 (*n* = 3)	60.7 ± 3.1 (*n* = 2)	76.4 ± 2.0 (*n* = 17)	66.3 ± 1.5 (*n* = 7)
9	64.3 (*n* = 1)		76.3 ± 2.3 (*n* = 8)	68.0 ± 1.1 (*n* = 3)
10			80.0 ± 2.6 (*n* = 4)	69.5 ± 1.5 (*n* = 2)
11			85.9 (*n* = 1)	

*Note*: The values are given as the mean and the standard deviation, *n* = sample size.

### 3.5 Growth patterns

The von Bertalanffy model was applied to both populations and fitted to the growth data for females and males (Table 3), indicating that mean body size increased with age. According to the results (Table 3), the predicted asymptotic body size of females was significantly larger than that of males in both populations because the confidence intervals did not overlap. There were no differences between the populations with predicted asymptotic body sizes for each sex. Females consistently had a higher mean growth rate than males across both populations. Additionally, the lower‐elevation population exhibited a higher mean growth rate than the higher‐elevation population (lower‐elevation: males, *R* = 5.64; females, *R* = 7.05; higher‐elevation: males, *R* = 3.49; females, *R* = 4.06).

**TABLE 3:** Growth parameters of von Bertalanffy's model for the Tibetan toad (*Bufo tibetanus*) at two elevational sites.

	Low elevation (2650 m)		High elevation (3930 m)	
	Estimate	95% CI	Estimate	95% CI
Males				
Asymptotic size (SVL_max_)	75.82	(71.24, 81.85)	76.08	(71.38, 82.3)
Growth coefficient (*k*)	0.23	(0.19, 0.28)	0.22	(0.18, 0.27)
Females				
Asymptotic size (SVL_max_)	94.76	(87.27, 105.57)	95.17	(87.48, 106.4)
Growth coefficient (*k*)	0.18	(0.14, 0.22)	0.17	(0.13, 0.22)

## 4 DISCUSSION

The present study applied skeletochronology to infer the individual ages of Tibetan toads from southwestern China. This technique has been widely used to estimate the age of amphibians inhabiting the Tibetan Plateau in China, enabling geographic comparisons of their life‐history patterns (Chen et al., 2011; Ma, Tong, & Lu, 2009; Yu et al., 2022). For these species, the formation of growth layers seems to be influenced by local climatic conditions, such as prevailing low temperatures for more than 4–5 months per year. Thus, bone growth can be affected by seasonal changes in weather. In general, bone grows rapidly during the active growth period (warm season) and slowly during the cold season. The toad normally hibernates between November and late April. Thus, the factors underlying the formation of the growth layers could be associated with the long inactive period of each year. In some cases, problems can arise due to the presence of double and false lines (Van Gelder & Hemelaar, 1979). However, this is less likely because the strong seasonal changes in temperature experienced by anurans rarely result in these lines (Chen et al., 2011; Ma, Lu, & Merilä, 2009; Yu et al., 2022).

The average body size of females was significantly larger than that of males in both populations. Female‐biased sexual size dimorphism is common among anurans and has traditionally been attributed to fecundity selection, favoring larger females that can lay more or larger eggs than small females (Han & Fu, 2013; Nali et al., 2014). This hypothesis has been supported by many studies which reported an increase in egg size with body size of the female for many anurans (Başkale et al., 2011; Hughes & Wylie, 2021; Pereira & Maneyro, 2012; Prado & Haddad, 2005; Zhao et al., 2017). Furthermore, sex differences in growth rates and age structures within populations can lead to sexual size dimorphism (Liao & Lu, 2012; Monnet & Cherry, 2002). Our data showed that females were larger than males only in the high‐elevation population after excluding the effect of age and that females were older than males in both populations. Therefore, age differences in both populations likely contributed to female‐biased sexual size dimorphism, as shown in studies on *Bufo hemiophrys* and *Rana swinhoana* (Eaton et al., 2005; Lai et al., 2005). Additionally, age‐specific growth rates may also play a role in the larger size of females at high elevations; however, further investigation is needed to confirm this. The lower age of males compared to females in both populations could result from the high costs of courtship displays, such as calling in breeding aggregations, which may expose them to greater predation risk (Sullivan & Kwiatkowski, 2007; Wells, 2001).

The morphological characteristics of the two Tibetan toad populations differed significantly, with the mean body size being larger in the higher‐elevation population. The SVL of both sexes fit the von Bertalanffy model well, indicating that the growth coefficient, asymptotic maximum size, metamorphic size, and age operate additively to determine post‐metamorphic body size (Liao & Lu, 2012). Because there was no significant difference in the growth coefficient and asymptotic maximum size for each sex between the two populations, the variation in body size must be explained by metamorphic size and age. The metamorphic size at the higher elevation was larger (lower elevation: 16.5 mm; higher elevation: 17.3 mm), and this may be an adaptive strategy for offspring survival through the cold winter, possibly achieved through females laying larger eggs (Liao et al., 2016; Morrison & Hero, 2003). The increased mean age of toads at higher elevations is possibly correlated with a decrease in the number of potential predators, as demonstrated in a lizard species (Ballinger, 1979). Moreover, individuals at low elevations may trade somatic growth for early reproduction in high‐mortality environments to avoid succumbing to predation before reproduction without leaving any offspring, resulting in a shorter lifespan (Williams et al., 2006).

The mean growth rates varied between the two populations, with a higher rate observed in the lower‐elevation population; however, these differences have not yet been statistically tested. Notably, these differences were not statistically significant. These results are similar to those of other studies on anuran species (Lai et al., 2005; Yu et al., 2022). According to the formula *R* = *k* (SVL_max_–SVL_mean_), the mean growth rate is affected by the growth coefficient, asymptotic maximum size, and mean body size. As shown in Table 3, neither the growth coefficient nor the asymptotic size differed significantly between the populations for either sex. Therefore, the observed variation in growth rate likely stemmed solely from the significant difference in the mean body size between the two populations.

The results showed that SVL positively correlated with age, but the size distributions among age classes overlapped extensively in both sexes, making age estimation from size data alone impossible in both populations. Similar results have been observed for many anuran species (Kyriakopoulou‐Sklavounou et al., 2008; Kyriakopoulou‐Sklavounou & Grumiro, 2002; Liao & Lu, 2010b; Yu et al., 2022; Zhang et al., 2020). In a few anuran species, a significant correlation between body size and age was found only in males (*Bufo pardalis*, Cherry & Francillon‐Vieillot, 1992) or females (*Rana temporaria*; Gibbons & McCarthy, 1984). For both sexes, the results of the rank‐based model showed that age was responsible for variations in body size. The body sizes of both sexes were larger in the higher‐elevation population, even when the effect of age was excluded, implying that Bergmann's rule applies to both sexes of Tibetan toads.

Overall, this study suggests that the age and body size differences in the Tibetan toad between populations could be affected by differences in environmental temperature, a pattern that is well‐documented in many anuran species (Hsu et al., 2014; Liao & Lu, 2011; Zhong et al., 2018). Except for the environmental temperature and length of the growing season, different elevations also exhibit notable variations in rainfall patterns, relative humidity, food availability, terrestrial microclimate, predation pressure, and other factors, all of which may affect the evolution of the life histories of anurans. Therefore, future research should prioritize disentangling the effects of these factors by studying populations in diverse environments.

## AUTHOR CONTRIBUTIONS

**Mengshuang Xu:** Data curation (lead); formal analysis (supporting); methodology (supporting); writing – original draft (supporting); writing – review and editing (equal). **Gege Wang:** Data curation (equal); writing – review and editing (equal). **Putong Liu:** Writing – review and editing (equal). **Zhuolin He:** Writing – review and editing (equal). **Kaiqin He:** Data curation (equal); writing – review and editing (equal). **Zhiqiang Cheng:** Writing – review and editing (equal). **Ziqi Wang:** Data curation (supporting); writing – review and editing (supporting). **Wei Chen:** Resources (equal); writing – review and editing (equal). **Zhibing Li:** Formal analysis (lead); software (lead); supervision (equal); writing – review and editing (equal). **Lixia Zhang:** Conceptualization (equal); formal analysis (equal); funding acquisition (lead); investigation (equal); methodology (lead); project administration (lead); resources (lead); software (equal); supervision (equal); validation (equal); visualization (lead); writing – original draft (lead); writing – review and editing (equal).

## CONFLICT OF INTEREST STATEMENT

The authors declare no competing interest.

### OPEN RESEARCH BADGES

This article has earned an Open Data badge for making publicly available the digitally‐shareable data necessary to reproduce the reported results. The data is available at [https://doi.org/10.6084/m9.figshare.24155142].

## Data Availability

The datasets supporting this article have been deposited in figshare https://doi.org/10.6084/m9.figshare.24155142.
